# Distinct clinicopathological characteristics, genomic alteration and prognosis in breast cancer with concurrent TP53 mutation and MYC amplification

**DOI:** 10.1111/1759-7714.14703

**Published:** 2022-10-28

**Authors:** Xiaoyi Lin, Xin Lin, Lijuan Guo, Yulei Wang, Guochun Zhang

**Affiliations:** ^1^ Department of Breast Surgery Guangdong Provincial People's Hospital, Guangdong Academy of Medical Sciences Guangzhou China; ^2^ Shantou University Medical College Shantou China; ^3^ The Second School of Clinical Medicine, Southern Medical University Guangzhou China; ^4^ School of Medicine, South China University of Technology Guangzhou China

**Keywords:** breast cancer (BC), MYC amplification, next‐generation sequencing (NGS), prognosis, TP53 mutation

## Abstract

**Background:**

Both TP53 mutation and MYC amplification indicate poor outcomes in breast cancer (BC), but the clinical values of concurrent TP53 and MYC alterations have not been well‐characterized.

**Methods:**

A total of 494 BC patients diagnosed at Guangdong Provincial People's Hospital (GDPH) were retrospectively analyzed. Genomic alterations were determined using next‐generation sequencing. Survival analysis was applied to assess the effects of genetic alterations on relapse‐free survival. The prognosis was verified based on 1405 patients from METABRIC cohort. Additionally, we used logistic regression to identify the factors associated with pathological complete response (pCR) after neoadjuvant chemotherapy.

**Results:**

In GDPH cohort, patients with TP53/MYC co‐alteration exhibited higher grade and stage, more positive HER2 status and higher Ki67 levels, but less luminal A subtypes. They also had more mutations in genes involved in ERBB and TGF‐β signaling pathways, as well as exclusive FANCG/CDKN2B/QKI copy number amplifications and SUFU/HIST3H3/ERCC4/JUN/BCR mutations. Concurrent TP53 and MYC alterations independently increased hazards of relapse (HR, 5.425; 95% CI: 2.019–14.579; *p* < 0.001). They maintained independent significance for relapse‐free (HR, 1.310; 95% CI: 1.012–1.697; *p* = 0.041) and overall survival (HR, 1.373; 95% CI: 1.093–1.725; *p* = 0.006) in METABRIC cohort. Among the 81 patients receiving chemotherapy, TP53 mutation (OR, 5.750; 95% CI: 1.553–25.776; *p* = 0.013) and earlier stage (OR, 0.275; 95% CI 0.088–0.788; *p* = 0.020) were associated with pCR, while the co‐alteration did not serve as an independent predictor (*p* = 0.199).

**Conclusions:**

TP53/MYC co‐alteration was associated with distinct clinicopathological and genomic features. They also conferred unfavorable prognosis in BC patients, and did not improve pCR after neoadjuvant chemotherapy.

## Graphical abstract

Breast cancer with concurrent TP53 mutation and MYC amplification demonstrated higher grade and stage, more positive HER2 status, higher Ki67 levels, and more mutations in genes involved in ERBB and TGF‐β signaling pathways, as well as exclusive FANCG/CDKN2B/QKI copy number amplifications. Additionally, TP53/MYC co‐alteration was associated with worse relapse‐free survival and overall survival in breast cancer patients.

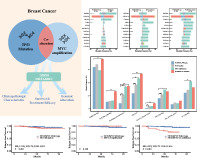

## INTRODUCTION

Breast cancer (BC), the most common female malignancy,^1^ is characterized by its biological heterogeneity.^2^, ^3^ Despite the declining mortality due to early diagnosis and various treatment strategies,^4^ the risks of BC recurrence and metastasis remain high.^5^, ^6^ Conventional tumor subtypes have been acknowledged as prognostic and therapeutic predictors.^7^, ^8^ However, with the development of high‐throughput next‐generation sequencing (NGS) technologies, comprehensive genomic landscapes have been delineated and more molecular events are being investigated.^9^, ^10^, ^11^ They play important roles in BC risk assessment and clinical decision‐making.

MYC, a common driver oncogene, has been reported to be amplified in approximately 15% of BCs.^12^ As a transcription factor, it not only enhances the expression of proliferation‐associated genes, but is also involved in DNA damage and genomic instability.^13^ Recently, it was reported that MYC could lead to palbociclib resistance in BC.^14^ TP53, a tumor suppressor that encodes p53 protein, was found to be mutated in about 45% of BCs.^3^ It cooperates with other transcription factors to inhibit growth and stimulate apoptosis.^15^ It is noteworthy that they are target genes of each other.^16^, ^17^ Previous studies determined that TP53 serves as a brake to control MYC‐mediated tumorigenesis, while mutant p53 activates the c‐MYC promoter.^17^, ^18^ Additionally, both TP53 mutation and MYC amplification increase glycolysis and anabolic metabolism, thus promoting cancer cell proliferation.^19^, ^20^ Numerous studies have revealed more common occurrence of these two alterations in triple‐negative BC (TNBC).^21^, ^22^ They confer a high risk of relapse and poor prognosis,^12^, ^23^, ^24^, ^25^ as well as endocrine therapy resistance.^26^

Nevertheless, the clinicopathological, genomic and prognostic significance of concurrent TP53 mutation and MYC amplification in BC has not been elucidated. In this context, we aimed to investigate whether concurrent TP53/MYC alteration correlates with distinct features, survival outcome and neoadjuvant chemotherapy response among BC patients.

## METHODS

### Patients and clinicopathological parameters

We retrospectively collected 494 BC patients with malignant diagnosis, DNA sequence data and follow‐up information at Guangdong Provincial People's Hospital (GDPH). Patients who were diagnosed with metastatic cancer or were not treatment naïve were excluded. Clinicopathological variables analyzed included age, pathological grade, histological type, stage, as well as hormone receptor (HR) and human epidermal growth factor receptor 2 (HER2) status assessed by the American Society of Clinical Oncology (ASCO)/College of American Pathologists (CAP) guidelines.^27^, ^28^ Ki67 expression levels were measured by IHC examination and recorded using the percentage of positive staining of malignant cells. 14% was the threshold to separate high/low expression groups. Based on the St Gallen International Expert Consensus criteria,^29^ IHC/FISH‐based surrogate molecular subtypes were determined according to estrogen receptor (ER), progestogen receptor (PR), HER2 status and Ki67 index.^7^, ^30^ Relapse‐free survival (RFS) was calculated from the date of first diagnosis to the date of first relapse or progression. BC cases who were free of relapse were censored at the date of last follow‐up visit.

### Next‐generation sequencing using a 520‐gene panel

In the present study, co‐alteration was defined if the BC sample were sequenced to harbor both TP53 mutation and MYC amplification. BC patients that had neither TP53 mutation nor MYC amplification, only TP53 mutation and only MYC amplification were defined as wild‐type (wt), TP53‐mut and MYC‐amp, respectively. Genomic results were obtained from NGS of breast tumors using a large 520‐gene panel. Detailed methods for NGS can be found in our previous research.^3^, ^31^ BC tissue samples were obtained through biopsy or surgery, and then processed into formalin‐fixed, paraffin‐embedded (FFPE) cell blocks. Tissue DNA was extracted from FFPE tumor blocks using QIAamp DNA FFPE tissue kit (Qiagen) and was measured using a Qubit 2.0 fluorimeter with dsDNA high sensitivity assay kit (Life Technologies). DNA was measured using Qubit 2.0 fluorimeter with dsDNA high sensitivity assay kit (Life Technologies). Later, tumor samples were sequenced using an Illumina NextSeq 500 instrument (Illumina Inc.) at Burning Rock Biotech, a Clinical Laboratory Improvement Amendments (CLIA)‐certified company in Guangzhou, China.

### Sequence data analysis

Sequence data were mapped to the reference human genome (hg19) using Burrows‐Wheeler Aligner version 0.7.10. Local alignment optimization, variant calling, and annotation were performed using GATK 3.2, MuTect, and VarScan. Single‐nucleotide variants were determined using at least eight supporting reads and SNP was determined based on the ExAC, 1000 Genomes, dbSNP, ESP6500SI‐V2 database. Variants without population frequency over 0.1% were annotated with ANNOVAR and SnpEff version 3.6. Copy number variation was assessed based on the depth of coverage data of capture intervals by in‐house analysis scripts. Coverage data were corrected, and the average coverage of all captured regions was used to normalize the coverage of different samples to comparable scales. Copy number was calculated based on the ratio between the depth of coverage in samples and average coverage of an adequate number (*n* > 50) of samples without copy number variation as references as to each capture interval. Copy number variation was determined if the coverage data of the gene region was quantitatively and statistically significantly different from its reference control, with a limit value of 2.64 for amplification.

### Neoadjuvant treatment efficacy

A total of 81 BC patients who were diagnosed at GDPH and received neoadjuvant chemotherapy were then selected. According to institutional guidelines, the chemotherapy regimens mainly included taxanes, epirubicin, doxorubicin, cyclophosphamide, 5‐fluorouracil, and carboplatin. Patients with positive HER2 status were treated with neoadjuvant chemotherapy plus HER2‐targeted therapy. In HER2‐ BC patients, the majority of patients (59.09%) received docetaxel/epirubicin/cyclophosphamide, while in HER2+ BC patients, most patients (83.78%) received docetaxel/carboplatin/trastuzumab. There were no differences in the distribution of other treatment options among different groups. Based on pathological review of a surgical specimen following chemotherapy, the absence of any residual invasive carcinoma in the breast and axilla was confirmed as pathological complete response (pCR). Logistic regression was used to evaluate the correlation of TP53 mutation and MYC amplification with neoadjuvant chemotherapy responses. Since the study cohort was heterogeneous, all clinicopathological factors above were selected into the univariable and multivariable logistic regression analysis to identify the independent risk factors.

### METABRIC cohort

Clinical and genomic data in Molecular Taxonomy of Breast Cancer International Consortium (METABRIC) were downloaded from cBio Cancer Genomics Portal.^32^ Stage I‐IV BC cases with complete histopathological information and follow‐up data were incorporated in METABRIC cohort. Group definition was the same as described above based on TP53/MYC alteration status. In addition to RFS, overall survival (OS) data was involved in the analysis, which was calculated as the time from diagnosis to death of any disease.

### Statistical analysis

Clinicopathological characteristics and mutation profiles of different subgroups were compared using chi‐square test or Fisher's exact test. Continuous variables were assessed by *t*‐test. Kaplan–Meier curves and log‐rank tests were utilized to compare the survival outcomes. Univariable and multivariable Cox proportional hazards regression models were used to evaluate the independent risk factor for prognosis. Two‐sided *p*‐values less than 0.05 were considered statistically significant. Statistical analysis was performed using R 4.1.2 software.

## RESULTS

### Patient characteristics

In this study, we included 494 BC patients with a median age of 47.5 years old. The association between clinicopathological features and TP53/MYC alteration is demonstrated in Table 1. The difference between MYC amplification and wild‐type groups was observed in diagnosis age. Patients diagnosed with MYC‐amp BC tended to be younger compared with those with wild‐type (median age, 42 vs. 47, *p* = 0.005). Both TP53 mutation and co‐alteration correlated with higher grade (grade III, TP53‐mut 70.79%, co‐alteration 66.67% vs. wt 27.78%), more advanced stage (stage III, TP53‐mut 22.77%, co‐alteration 30.77% vs. wt 14.53%), positive HER2 status (TP53‐mut 50.00%, co‐alteration 41.03% vs. wt 16.67%) and high Ki67 index (TP53‐mut 91.09%, co‐alteration 94.87% vs. wt 63.25%). Negative HR status was more common in TP53‐mut patients (39.11%) but did not exhibit significant difference between co‐alteration and the wild‐type group (15.38% vs. 9.40%). IHC/FISH‐based molecular subtype distributions were significantly different in all groups compared with the wild‐type group, especially for TP53‐mut and co‐alteration. Luminal B (HER2+) subtypes made up a markedly greater proportion in the co‐alteration group than the wild‐type (33.33% vs. 12.39%), while luminal A subtypes were much rarer (0.00% vs. 29.49%). Both HER2‐enriched (TP53‐mut 20.79%, co‐alteration 7.69% vs. wt 4.27%) and TNBC subtypes (TP53‐mut 17.82%, co‐alteration 7.69% vs. wt 5.13%) were more common in the TP53‐mut and co‐alteration groups.

**TABLE 1:** Baseline characteristics of GDPH BC patients

	TP53/MYC wild‐type	TP53 mutation	MYC amplification	Co‐alteration			
Variables	(N = 234)	(N1 = 202)	(N2 = 19)	(N3 = 39)	P1	P2	P3
Age at diagnosis					0.238	0.005	0.360
Median (range)	47 (25–85)	48 (22–79)	42 (35–60)	48 (23–65)			
Grade (%)					<0.001	0.515	<0.001
I	15 (6.41%)	1 (0.50%)	0 (0.00%)	0 (0.00%)			
II	154 (65.81%)	58 (28.71%)	13 (68.42%)	13 (33.33%)			
III	65 (27.78%)	143 (70.79%)	6 (31.58%)	26 (66.67%)			
Histological type (%)					0.232	1	0.378
IDC	204 (87.18%)	179 (88.61%)	18 (94.74%)	37 (94.87%)			
ILC	8 (3.42%)	2 (0.99%)	0 (0.00%)	1 (2.56%)			
Others	22 (9.40%)	21 (10.40%)	1 (5.26%)	1 (2.56%)			
Stage (%)					0.040	0.883	0.030
I	62 (26.50%)	39 (19.31%)	5 (26.32%)	6 (15.38%)			
II	138 (58.97%)	117 (57.92%)	12 (63.16%)	21 (53.85%)			
III	34 (14.53%)	46 (22.77%)	2 (10.53%)	12 (30.77%)			
HR status (%)					<0.001	0.329	0.393
Negative	22 (9.40%)	79 (39.11%)	0 (0.00%)	6 (15.38%)			
Positive	212 (90.60%)	123 (60.89%)	19 (100.00%)	33 (84.62%)			
HER2 status (%)					<0.001	0.071	0.001
Negative	187 (79.91%)	95 (47.03%)	14 (73.68%)	21 (53.85%)			
Positive	39 (16.67%)	101 (50.00%)	2 (10.53%)	16 (41.03%)			
Equivocal	8 (3.42%)	6 (2.97%)	3 (15.79%)	2 (5.13%)			
Ki67 expression (%)					<0.001	0.053	<0.001
Median	20	40	25	40			
>14	148 (63.25%)	184 (91.09%)	17 (89.47%)	37 (94.87%)			
≤14	84 (35.90%)	17 (8.42%)	2 (10.53%)	2 (5.13%)			
Unknown	2 (0.85%)	1 (0.50%)	0 (0%)	0 (0%)			
IHC/FISH‐based molecular subtype (%)					<0.001	0.027	<0.001
Lum A	69 (29.49%)	7 (3.47%)	1 (5.26%)	0 (0%)			
Lum B (HER2‐)	105 (44.87%)	51 (25.25%)	13 (68.42%)	18 (46.15%)			
Lum B (HER2+)	29 (12.39%)	59 (29.21%)	2 (10.53%)	13 (33.33%)			
HER2‐enriched	10 (4.27%)	42 (20.79%)	0 (0%)	3 (7.69%)			
TNBC	12 (5.13%)	36 (17.82%)	0 (0%)	3 (7.69%)			
Unknown	9 (3.85%)	7 (3.47%)	3 (15.79%)	2 (5.13%)			

Abbreviations: BC, breast cancer; Co‐alteration, TP53 mutation and MYC amplification; GPDH, Guangdong Provincial People's Hospital; HER2 status, human epidermal growth factor receptor 2 status; HR status, hormone receptor status; IDC, infiltrating ductal carcinoma; ILC, infiltrating lobular carcinoma; Lum A, luminal A; Lum B, luminal B; P1, TP53 mutation vs. wild‐type based on chi‐square or Fisher's exact test; P2, MYC amplification vs. wild‐type; P3, co‐alteration vs. wild‐type; TNBC, triple‐negative BC.

### Mutation landscape

**FIGURE 1:**
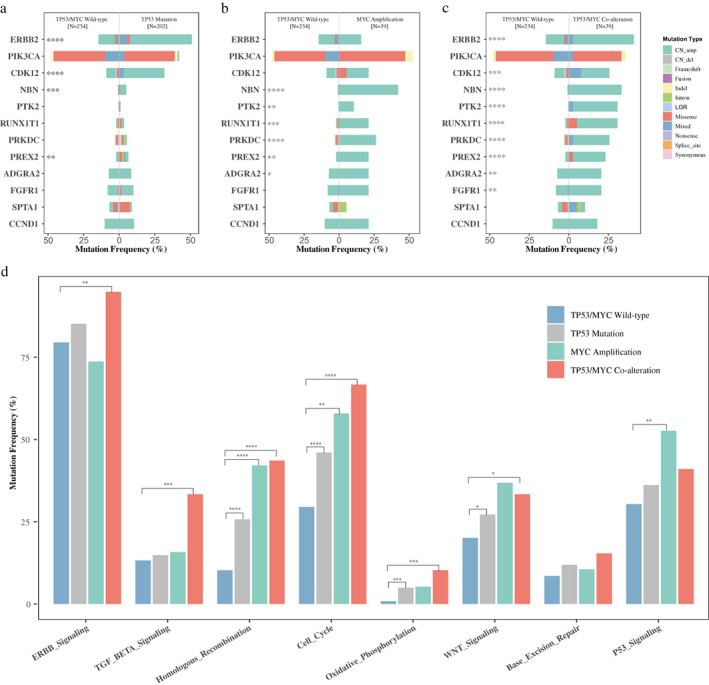
Differentially mutated genes and molecular signaling pathways. (a) Gene mutation frequency between breast cancer (BC) patients with TP53 mutation and without TP53/MYC alteration. (b) Gene mutation frequency between BC patients with MYC amplification and without TP53/MYC alteration. (c) Gene mutation frequency between BC patients with TP53/MYC co‐alteration and without TP53/MYC alteration. (d) Mutation frequency of genes involved in molecular pathways in BC patients. The asterisk (*) indicated statistically significant difference between different groups based on chi‐square or Fisher's exact test: *p < 0.1, **p < 0.05, ***p < 0.01, ****p < 0.001

Based on the genomic results from 520‐gene panel sequencing, FANCG, CDKN2B, QKI copy number amplifications, and SUFU, HIST3H3, ERCC4, JUN, BCR mutations were exclusively detected together with TP53 mutation and MYC amplification. It is notable that two samples in the co‐alteration group harbored contaminant FANCG and CDKN2B amplifications. Then, we analyzed the association between TP53/MYC alteration status and mutation frequency of the 12 most commonly altered genes (except TP53 and MYC) in the co‐alteration group (Figure 1(a–c)). Patients diagnosed with contaminant TP53 mutation and MYC amplification had significantly more mutations in ERBB2 (*p* < 0.001), CDK12 (*p* = 0.006), NBN (*p* < 0.001), PTK2 (*p* < 0.001), RUNX1T1 (*p* < 0.001), PRKDC (*p* < 0.001), PREX2 (*p* < 0.001), ADGRA2 (*p* = 0.018) and FGFR1 (*p* = 0.035) as compared with patients in the wild‐type group. TP53‐mut BCs were detected with significantly more mutations in ERBB2 (*p* < 0.001), CDK12 (*p* < 0.001), NBN (*p* = 0.009) and PREX2 (*p* = 0.024) than the wild‐type, while MYC‐amp BC had significantly higher mutation rates in NBN (*p* < 0.001), PTK2 (*p* = 0.015), RUNX1T1 (*p* = 0.002), PRKDC (*p* < 0.001) and PREX2 (*p* = 0.016).

The mutation frequency of genes (except TP53 and MYC) associated with molecular pathways of interest is demonstrated in Figure 1(d). Among the BC patients with TP53/MYC co‐alteration, 37 (94.87%) had mutations in genes in ERBB signaling pathway, 26 (66.67%) had mutations in genes involved in the cell cycle, 17 (43.59%) had mutations in genes involved in homologous recombination, and 16 (41.03%) had mutations in genes in the p53 signaling pathway. Patients with co‐alteration had significantly more mutations in genes involved in the ERBB signaling pathway (*p* = 0.021), TGF‐β signaling pathway (*p* = 0.002), homologous recombination (*p* < 0.001), cell cycle (*p* < 0.001), and oxidative phosphorylation (*p* = 0.004) as compared with the wild‐type group. More alterations in genes involved in homologous recombination (*p* < 0.001), cell cycle (*p* < 0.001), and oxidative phosphorylation (*p* = 0.004) in TP53‐mut patients, and more mutations in genes involved in homologous recombination (*p* < 0.001), cell cycle (*p* = 0.01) and the p53 signaling pathway (*p* = 0.045) in MYC‐amp patients were detected when compared with BC patients without TP53/MYC alteration.

### Prognostic effects of TP53 mutation and MYC amplification

**FIGURE 2:**
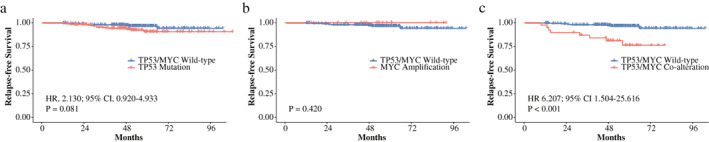
Kaplan–Meier curves of relapse‐free survival (RFS) among breast cancer (BC) patients stratified by TP53/MYC alterations. (a) RFS rates between BC patients harboring TP53 mutation and without TP53/MYC alteration. (b) RFS rates between BC patients harboring MYC amplification and without TP53/MYC alteration. (c) RFS rates between BC patients harboring co‐alteration and without TP53/MYC alteration

Of the eligible patients, the median follow‐up period was 50.55 months. Relapse occurred in eight patients (3.42%) in the TP53/MYC wild‐type group, 14 patients (6.93%) in the TP53‐mut group, and eight patients (20.51%) in the co‐alteration group. BC patients with TP53‐mut had an almost nonsignificant shorter RFS (HR, 2.130; 95% CI: 0.920–4.933; *p* = 0.081) than those without TP53/MYC alteration (Figure 2(a)). As shown in Figure 2(c), the distinction was significant when co‐alteration group was compared with the wild‐type group (HR, 6.207; 95% CI: 1.504–25.616; *p* < 0.001). No difference was found between MYC‐amp and wild‐type BC patients (*p* = 0.420) (Figure 2(b)). In univariable Cox regression analysis, co‐alteration (univariable HR, 6.203; 95% CI: 2.326–16.539; *p* < 0.001) was associated with increased hazards of relapse. After incorporating aforementioned variables (age, grade, stage, HR, HER2 and Ki67) into the univariable analysis, stage appeared to correlate with RFS (stage III vs. stage I, univariable HR, 2.954; 95% CI: 1.051–8.299; *p* = 0.040). However, only concurrent TP53 mutation and MYC amplification maintained significance for RFS in multivariable Cox regression model (multivariable HR, 5.425; 95% CI: 2.019–14.579; *p* < 0.001) (Table 2). Therefore, this co‐alteration independently indicated poor RFS in GDPH BC patients.

**TABLE 2:** Univariable and multivariable Cox regression analysis of RFS in GDPH BC patients

	Univariable analysis		Multivariable analysis	
Variables	HR (95% CI)	*p*	HR (95% CI)	*p*
Stage				
I	1		1	
II	0.843 (0.297, 2.398)	0.749	0.793 (0.278, 2.257)	0.663
III	2.954 (1.051, 8.299)	0.040	2.448 (0.865, 6.928)	0.092
TP53/MYC status				
TP53/MYC wild‐type	1		1	
TP53 mutation	2.125 (0.891, 5.067)	0.089	1.944 (0.811, 4.657)	0.136
MYC amplification	0.000 (0, +∞)	0.997	0.000 (0, +∞)	0.997
Co‐alteration	6.203 (2.326, 16.539)	<0.001	5.425 (2.019, 14.579)	<0.001

Abbreviations: BC, breast cancer; CI, confidence interval; Co‐alteration, TP53 mutation and MYC amplification; GDPH, Guangdong Provincial People's Hospital; HR, hazard ratio; RFS, relapse‐free survival.

### Relapse‐free survival and overall survival in METABRIC cohort

**FIGURE 3:**
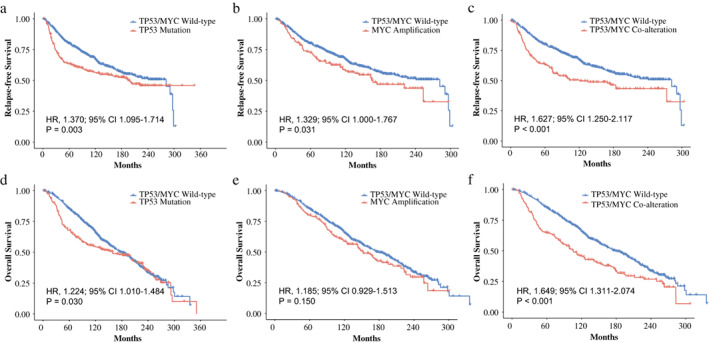
Relapse‐free survival (RFS) and overall survival (OS) outcomes of breast cancer (BC) patients according to TP53/MYC alteration in METABRIC cohort. (a) RFS rates between BC patients harboring TP53 mutation and without TP53/MYC alteration. (b) RFS rates between BC patients harboring MYC amplification and without TP53/MYC alteration. (c) RFS rates between BC patients harboring co‐alteration and without TP53/MYC alteration. (d) OS rates between BC patients harboring TP53 mutation and without TP53/MYC alteration. (e) OS rates between BC patients harboring MYC amplification and without TP53/MYC alteration. (f) OS rates between BC patients harboring co‐alteration and without TP53/MYC alteration.

To confirm the prognostic roles of TP53/MYC co‐alteration, 1405 BC cases in METABRIC cohort were further analyzed. In this cohort, 196 (13.95%), 272 (19.36%) and 156 (11.10%) patients harbored co‐alteration, only TP53 mutation and only MYC amplification, respectively. According to the Kaplan–Meier curves in Figure 3(a–c), RFS was worse for patients harboring TP53 mutation or MYC amplification, in comparison with wild‐type BC patients. It was most prominent for the discrepancy between co‐alteration and the wild‐type group (HR, 1.627; 95% CI: 1.250–2.117; *p* < 0.001). In Figure 3(d), OS was significantly shorter in patients with TP53‐mut than in the wild‐type group (HR, 1.224; 95% CI: 1.010–1.484; *p* = 0.030), but was similar between MYC‐amp and wild‐type groups (*p* = 0.150) (Figure 3(e)). The 5‐year OS rates were 85.0% (95% CI: 82.5%–87.6%) and 64.5% (95% CI: 58.1%–71.6%) in the wild‐type and co‐alteration groups, respectively. As Figure 3(f) exhibited, co‐alteration showed a tendency towards shorter survival than that of wild‐type BC (HR, 1.649; 95% CI: 1.311–2.074; *p* < 0.001).

Subsequent univariable Cox regression analysis revealed TP53/MYC alteration (TP53‐mut vs. wild‐type, HR 1.377; 95% CI: 1.118–1.697; *p* = 0.003; MYC‐amp vs. wild‐type, HR 1.314; 95% CI: 1.013–1.703; *p* = 0.039; co‐alteration vs. wild‐type, HR 1.613; 95% CI: 1.283–2.029; *p* < 0.001), as well as higher grade (II vs. I, HR 1.526; 95% CI: 1.047–2.225; *p* = 0.028; III vs. I, HR 2.116; 95% CI: 1.465–3.056; *p* < 0.001), higher stage (II vs. I, HR 1.597; 95% CI: 1.321–1.930; *p* < 0.001; III vs. I, HR 3.434; 95% CI: 2.603–4.530; *p* < 0.001; IV vs. I, HR 9.040; 95% CI: 4.758–17.170; *p* < 0.001), negative HR status (HR 1.223; 95% CI: 1.012–1.477; *p* = 0.037), positive HER2 status (HR 1.757; 95% CI: 1.415–2.181; *p* < 0.001) as potential risk factors for RFS. After adjusting for the confounders, concurrent TP53 mutation and MYC amplification retained the independent, unfavorable impact on RFS (multivariable HR, 1.310; 95% CI: 1.012–1.697; *p* = 0.041). Regarding OS, consistently, the co‐alteration was proved to be an independent prognostic factor for BC patients by Cox regression models (univariable HR, 1.635; 95% CI: 1.342–1.992; *p* < 0.001; multivariable HR, 1.373; 95% CI: 1.093–1.725; *p* = 0.006). The results of multivariable analyses in METABRIC cohort are presented in Table 3.

**TABLE 3:** Multivariable Cox regression modeling results of RFS and OS among BC patients in METABRIC cohort

	Multivariable analysis for RFS		Multivariable analysis for OS	
Variables	HR (95% CI)	*p*‐value	HR (95% CI)	*p*‐value
Grade				
I	1		1	
II	1.304 (0.892, 1.908)	0.171	1.141 (0.845, 1.540)	0.388
III	1.574 (1.068, 2.320)	0.022	1.270 (0.933, 1.729)	0.128
Stage				
I	1		1	
II	1.509 (1.246, 1.827)	<0.001	1.73 (1.468, 2.040)	<0.001
III	3.087 (2.332, 4.086)	<0.001	3.066 (2.370, 3.967)	<0.001
IV	9.680 (5.070, 18.481)	<0.001	6.459 (3.289, 12.682)	<0.001
HR status				
Negative	1		1	
Positive	1.166 (0.931, 1.462)	0.181	1.108 (0.905, 1.356)	0.322
HER2 status				
Negative	1		1	
Positive	1.539 (1.222, 1.940)	<0.001	1.365 (1.103, 1.69)	0.004
TP53/MYC status				
TP53/MYC wild‐type	1		1	
TP53 mutation	1.191 (0.936, 1.514)	0.155	1.106 (0.894, 1.367)	0.354
MYC amplification	1.168 (0.897, 1.521)	0.249	1.086 (0.859, 1.373)	0.491
Co‐alteration	1.310 (1.012, 1.697)	0.041	1.373 (1.093, 1.725)	0.006

Abbreviations: BC, breast cancer; CI, confidence interval; Co‐alteration, TP53 mutation and MYC amplification; HER2 status, human epidermal growth factor receptor 2 status; HR status, hormone receptor status; HR, hazard ratio; OS, overall survival; RFS, relapse‐free survival.

### Correlation of co‐alteration with therapeutic efficacy

In the GDPH cohort, 81 BC patients received neoadjuvant chemotherapy, among which 30 patients (37.04%) achieved PCR. In univariable logistic regression including all the known clinicopathological variables (Table 4), later stage (stage III vs. II, OR, 0.350; 95% CI: 0.132–0.881; *p* = 0.029) and negative HER2 status (HER2‐ vs. HER2+, OR, 0.250; 95% CI: 0.093–0.637; *p* = 0.005) were risk factors for pCR. Patients with TP53‐mut BC were more likely to reach pCR (OR, 5.250; 95% CI: 1.646–20.507; *p* = 0.009) as compared to wild‐type patients. However, concurrent TP53 mutation and MYC amplification did not show a higher likelihood of achieving pCR (OR, 3.150; 95% CI: 0.738–14.864; *p* = 0.127). The independent impacts of stage (stage III vs. II, OR, 0.275; 95% CI: 0.088–0.788; *p* = 0.127) and TP53 mutation (OR, 3.150; 95% CI: 0.738–14.864; *p* = 0.127) were verified by multivariable logistic analysis.

**TABLE 4:** Logistic regression analysis of pCR in GDPH BC patients

	Univariable analysis		Multivariable analysis	
Variables	Odds ratio (95% CI)	*p*‐value	Odds ratio (95% CI)	*p*‐value
Stage				
II	1		1	
III	0.350 (0.132, 0.881)	0.029	0.275 (0.088, 0.788)	0.020
HER2				
Positive	1		1	
Negative	0.250 (0.093, 0.637)	0.005	0.359 (0.125, 0.996)	0.052
TP53/MYC status				
TP53/MYC wild‐type	1		1	
TP53 mutation	5.250 (1.646, 20.507)	0.009	5.750 (1.553, 25.776)	0.013
Co‐alteration	3.150 (0.738, 14.864)	0.127	2.849 (0.585, 15.135)	0.199

Abbreviations: BC, breast cancer; CI, confidence interval; Co‐alteration, TP53 mutation and MYC amplification; GDPH, Guangdong Provincial People's Hospital; OR, odds ratio; pCR, pathological complete response.

## DISCUSSION

TP53 mutation and MYC amplification played vital roles in BC prognosis and treatment options.^23^, ^26^ TP53/MYC co‐alteration accounted for a substantial proportion of BC patients.^3^, ^22^ Thus, research on its correlation with clinical outcomes is urgently required.

In the GDPH cohort, we revealed the differences at the clinicopathological and molecular level in BCs with concurrent TP53 and MYC alteration. The co‐alteration was associated with high grade, advanced stage, positive HER2 status, high Ki67 levels, IHC/FISH‐based luminal B (HER2+) subtypes. FANCG, CDKN2B, QKI copy number amplifications, and SUFU, HIST3H3, ERCC4, JUN, BCR mutations were exclusively detected in BCs with co‐alteration. They were detected with significantly more mutations in ERBB2, CDK12, NBN, PTK2, RUNX1T1, PRKDC, PREX2, ADGRA2, FGFR1 and more alterations in genes involved in ERBB signaling pathway, TGF‐β signaling pathway, homologous recombination, cell cycle, and oxidative phosphorylation. Furthermore, we found that concurrent TP53 mutation and MYC amplification suggested worse RFS. In METABRIC cohort, the RFS and OS were both shorter in patients harboring co‐alteration. This finding could be explained by their synergistic effects. As a safeguard, p53‐independent DNA damage response was activated to prevent MYC‐mediated tumorigenesis.^18^, ^33^, ^34^ Reversely, deregulated MYC partially disabled the p53‐mediated apoptosis, therefore leading to poor clonogenic survival.^35^ Both TP53 mutation and MYC amplification were associated with aneuploidy, and thus genomic instability and intratumor heterogeneity.^36^ According to previous reports, they were independent worse prognostic factors in BC,^12^, ^23^, ^37^ mostly in metastatic BC or certain molecular subtypes.^24^, ^26^, ^38^, ^39^, ^40^ Nonetheless, neither TP53 mutation nor MYC amplification alone was independently correlated with poor prognosis in our study. It was probably because of differences in race and samples composition, including Chinese and western population as well as the short‐ and long‐term follow‐up period. Moreover, the confounding factors, such as stages, HR and HER2 status were taken into account to reach a reliable conclusion.

Previous studies have proposed pCR after neoadjuvant therapy as a surrogate endpoint for better survival among BC patients.^41^, ^42^ In our study, TP53 mutation was independently correlated with better pCR after neoadjuvant chemotherapy. However, when combined with MYC amplification, the predictive value no longer existed. TP53 mutation could lead to rapid tumor clearance by DNA‐damaging therapies, due to loss of p53‐enforced cell cycle checkpoint and transcriptional control.^43^ Expectedly, consistent with existing research,^44^, ^45^ higher pCR rate in TP53‐mut BC patients was observed. But the effect of MYC amplification remained controversial. Pereira et al. reported the role of MYC amplification in chemosensitivity,^46^ and Yasojima et al. restricted this correlation to ER‐positive BC.^47^ It seemed that the effect of TP53 mutation in chemotherapy response of BC was weakened by the concurrent MYC amplification.

Although we comprehensively depicted the prognosis and neoadjuvant chemotherapy response of the TP53/MYC co‐alteration in BC, there are some limitations in our study. The samples in GDPH cohort were too small to draw a rigorous conclusion. Although a public database was utilized to confirm our results, larger samples are required to verify the clinical validity and usefulness of co‐alteration as a novel biomarker. In addition, there were some differences in neoadjuvant therapeutic regimens, and more homogeneous analyses are therefore necessary in the future.

In conclusion, there were some significant differences in the clinicopathological and genomic characteristics for BCs with concurrent TP53 mutation and MYC amplification. Concurrent TP53/MYC alteration also portended dismal prognosis in BC survivors. Although this co‐alteration could serve as an independent prognostic factor, it was not able to be a predictor of neoadjuvant chemotherapy response.

## CONFLICT OF INTEREST

The authors declare that they have no conflict of interest.
